# *Citrus bergamia* essential oil: from basic research to clinical application

**DOI:** 10.3389/fphar.2015.00036

**Published:** 2015-03-02

**Authors:** Michele Navarra, Carmen Mannucci, Marisa Delbò, Gioacchino Calapai

**Affiliations:** ^1^Department of Drug Sciences and Products for Health, University of MessinaMessina, Italy; ^2^Department of Clinical and Experimental Medicine, University of MessinaMessina, Italy; ^3^Italian Medicines AgencyRome, Italy

**Keywords:** *Citrus bergamia*, bergamot essential oil, natural products, complementary and alternative medicine, aromatherapy

## Abstract

*Citrus bergamia* Risso et Poiteau, also known as “Bergamot,” is a plant belonging to the Rutaceae family, defined as a hybrid of bitter orange and lemon. It is an endemic plant of the Calabria region (Italy). Bergamot fruit is primarily used for the extraction of its essential oil (bergamot essential oil: BEO), employed in perfume, cosmetics, food, and confections. The aim of this review was to collect recent data from the literature on *C. bergamia* essential oil and, through a critical analysis, focus on safety and the beneficial effects on human health. Clinical studies on the therapeutic applications of BEO exclusively focus on the field of aromatherapy, suggesting that its use can be useful for reducing anxiety and stress.

## INTRODUCTION

Bergamot is the common name for *Citrus bergamia* Risso et Poiteau, a plant belonging to the Rutaceae family (subfamily Esperidea). The trees show big dark green ovate leaves similar to those of lemon, star shaped white flowers and round yellow fruits. The botanical and geographical origins of this plant are still uncertain (Rapisarda and Germanò, 2013). It may be native of the Calabria region (Italy), as a result of mutations from other species. Alternatively, it may originate from Antilles, Greece, and the Canary Islands, from where Christopher Columbus imported it. The name “bergamot” seems to be derived from Berga, the Spanish city from which the plant was transported in Calabria (south of Italy) later. *C. bergamia* trees are cultivated almost exclusively along the southern coast of the Calabria region (more than 90% of the world production of bergamot comes from this region). However, small numbers of bergamot plants grow in other countries, such as Greece, Morocco, Iran and Ivory Coast, Argentina, and Brazil.

*Citrus bergamia* is defined as a hybrid between a sour orange (*C. aurantium* L.) and lemon (*C. limon* L. Burm. f.) or a mutation of the latter. Other authors considered it a hybrid between a sour orange and lime (*C. aurantifolia* [Christm. and Panzer] Swingle; Rapisarda and Germanò, 2013). Bergamot fruit is mainly used for its essential oils (bergamot essential oil: BEO) that are obtained by rasping and cold pressing the fruit peel. BEO is widely used in perfume, cosmetics, food, and confectionery industries for its intense fragrance and freshness. BEO is a greenish or brownish-yellow volatile oil (corresponding to the beginning and the end of the productive season) with a bitter aromatic taste and a characteristic pleasant odor. It is included in various countries’ official Pharmacopoeias. However, bergamot juice, obtained from the endocarp after essential oils extraction, has long time been considered just a secondary and discarded product of the essential oil industry due to its bitter taste. Recently, it gained attention because of its hypolipemic and hypoglycaemic activity (Mollace et al., 2011), as well as its anti-inflammatory (Impellizzeri et al., 2014; Risitano et al., 2014) and anti-cancer properties (Delle Monache et al., 2013; Navarra et al., 2014; Visalli et al., 2014).

## BEO CHEMICAL COMPOSITION

The chemical composition of BEO has been widely investigated and is well known (Costa et al., 2010; Dugo and Bonaccorsi, 2013). BEO contains several bioactive molecules with potential health benefits. It is composed of both a volatile (93–96% of total) and a non-volatile (4–7% of total) fraction. The first is mainly represented by monoterpene and sesquiterpene hydrocarbons, and their oxygenated derivatives, along with aliphatic aldehydes, alcohols, and esters (Dugo and Bonaccorsi, 2013). They include monoterpene limonene (25–53%) and high quantities of oxygenated compounds, such as linalool (2–20%), linalyl acetate (15–40%), γ-terpinene, and β-pinene (Mondello et al., 1998). The non-volatile fraction (4–7% of total) contains pigments, waxes, coumarins, and psoralens (such as 5-methoxypsoralen, also known as bergapten or 5-MOP, contained in about 0.2%), as well as bergamottine [5-geranyloxypsoralen]) (Dugo et al., 2000). Due to the well-known 5-MOP-induced photo-toxicity, a furocoumarins-free essential oil has been prepared for perfumery and cosmetic uses. The vacuum distillation of bergamot peels provides a high-quality BEO totally devoid of 5-MOP that is chemically comparable to that of the cold-pressed oil (Belsito et al., 2007).

The characteristic flavor of *Citrus* oils is mainly provided by linalool, citral, and linalyl acetate, (Fang et al., 2004), whereas limonene and pinene are not much flavoring and they are relatively unstable compounds when exposed to heat and light. Thus, it is necessary to remove them to increase the shelf life of the products (Reverchon and Iacuzio, 1997; Fang et al., 2004).

## BEO INDUSTRIAL AND MEDICINAL USES

Bergamot essential oil is one of the main basic constituents for the manufacture of perfumes, due to its ability to fix the aromatic bouquet of aromas and harmonize all of the essences, enhancing the fragrance. BEO is also used by the pharmaceutical industry, both to absorb the unpleasant smells of medicinal products and for its antiseptic and antibacterial properties. Finally, BEO is used in the food and confectionery industries as a flavoring.

In Italian folk medicine, it has been used primarily for fever and parasitic diseases, in addition to mouth, skin, respiratory and urinary tract infections, gonococcal infections, leucorrhoea, vaginal pruritus, tonsillitis, and sore throats (Pendino, 1998). For its antiseptic and antibacterial proprieties, BEO has been used as an antimicrobial agent to facilitate wound healing and has been included in preparations used to treat upper respiratory-tract disorders and hyperhidrosis. Moreover, BEO’s use in magisterial, handcrafted, and homemade medicaments that were useful for skin disinfection, as well as use as an aid for healing minor wounds, has a long tradition in Italy. Currently, a furocoumarins-free BEO is used in preparations for cutaneous use.

Bergamot essential oil is widely employed in aromatherapy, and has recently received renewed popularity in improving mood and mild symptoms of stress-induced disorders (Halcon, 2002) and facilitating sleep induction (Wiebe, 2000). Aromatherapy massage has been shown to relieve symptoms of anxiety in patients with cancer (Wilkinson et al., 2007).

## BIOLOGICAL ACTIVITIES OF BEO

### ANTIMICROBIAL ACTIVITY

It has been reported that BEO has both antibacterial and antifungal activity against *Campylobacter jejuni*, *Escherichia coli* O157, *Listeria monocytogenes*, *Bacillus cereus*, and *Staphylococcus aureus* and dermatophytes, respectively (Karaca et al., 2007). The *in vitro* activity of BEO against *Candida species* suggests BEO’s potential role in the topical treatment of *Candida infections* (Romano et al., 2005). BEO is also active against dermatophytes *in vitro* (Sanguinetti et al., 2007). Additionally, chitosan-based films containing BEO at 0.5, 1, 2, and 3% w/w showed a significant dose-dependent inhibitory effect on the growth of *Penicillium italicum* (Sánchez-González et al., 2010). Moreover, the *in vitro* effectiveness of the oil and vapors of bergamot and its components against common foodborne pathogens has been also investigated, and linalool was revealed to be the most effective anti-bacterial component (Fisher and Phillips, 2006).

### ANTI-INFLAMMATORY ACTIVITY

Bergamot essential oil anti-inflammatory activity was demonstrated using the carrageenan-induced rat paw oedema test. The highest level of BEO anti-inflammatory activity was obtained with a 0.10 ml/kg dosage. The median effective dose of BEO was found to be 0.079 ml/kg (Karaca et al., 2007).

### ANTIPROLIFERATIVE ACTIVITY

Bergamot essential oil has also been found to inhibit the survival and proliferation of SH-SY5Y neuroblastoma cells, (Celia et al., 2013) through the activation of multiple pathways leading to both necrotic and apoptotic cell death (Ursino et al., 2010). Moreover, Russo et al. (2013) showed that association of limonene and linalyl acetate, but not the exposure to the single compounds, caused significant cytotoxicity, suggesting for a major role of the combined action of these monoterpenes in cancer cell death induced by BEO.

### NEUROPSYCHOPHARMACOLOGICAL AND NEUROPROTECTIVE ACTIVITIES

Anxiolytic effects of BEO (1.0, 2.5, and 5.0% w/w) were studied by administering it to rats subjected to anxiety-related behaviors, the elevated plus-maze and the hole-board tests, and then measuring the stress-induced levels of plasma corticosterone in comparison with the effects of diazepam. BEO (2.5%) and diazepam exhibited anxiolytic-like effects and attenuated the corticosterone response to acute stress (Saiyudthong and Marsden, 2010).

After perfusion into the hippocampus via the dialysis probe (20 μl/20 min), BEO produced a dose-dependent and Ca^2+^-independent increase of extracellular aspartate, glycine, taurine, GABA, and glutamate (Morrone et al., 2007). Moreover, BEO (0.5 ml/kg) given intraperitoneally 1 h before experimental occlusion of the middle cerebral artery, significantly reduced infarct size after 24 h, especially in the medial striatum and the motor cortex, as revealed by 2,3,5-triphenyl-2H-tetrazolium chloride (TTC) staining of tissue slices (Amantea et al., 2009).

Finally, in the human SH-SY5Y neuroblastoma cell line exposed to *N*-methyl-D-aspartate (NMDA), BEO (0.0005–0.01%) reduced the death of SH-SY5Y cells caused by 1 mM NMDA in a concentration dependent manner. In addition, 0.01% BEO counteracted the deactivation of Akt (a serine/threonine-specific protein kinase) and the consequent activation of glycogen synthase kinase 3 beta (GSK-3B) induced by NMDA. Results obtained with specific fractions of BEO, suggested that monoterpene hydrocarbons could be important for neuroprotection (Corasaniti et al., 2007).

### ANALGESIC EFFECTS

Capsaicin-induced nociceptive responses in the plantar surface of the hindpaw were significantly reduced by intraplantar injection of BEO. Further experiments addressed the importance of linalool in BEO oil-induced antinociception (Sakurada et al., 2009). In another study, injection into the hindpaw of both the linalool and linalyl acetate compounds showed a significant reduction of nociceptive responses, which was much more potent than that induced by BEO. The enhanced effect of the association of BEO or linalool with morphine was antagonized by pretreatment with the opioid antagonist naloxone (Sakurada et al., 2011). The analgesic effect was also investigated using intraplantar injection of BEO or linalool in mice with neuropathic hypersensitivity induced by partial sciatic nerve ligation (PSNL). The results suggested that both BEO and linalool reduced PSNL-induced mechanical allodynia in a dose-dependent manner through a local effect. Moreover, the analgesic effect was associated with a reduction of spinal extracellular signal-regulated protein kinase (ERK) activation (Kuwahata et al., 2013).

### CARDIOVASCULAR PROPERTIES

Bergamottine significantly decreased the electrocardiographic changes that are typical of coronary arterial spasms and the occurrence of experimental cardiac arrhythmias provoked by pitressin in guinea pigs. Bergamottine also increased the dose of ouabain required to cause ventricular premature beats, ventricular tachyarrhythmias, and death. These results indicate that bergamottine possesses potential antianginal and antiarrhythmic properties (Occhiuto and Circosta, 1996).

It is known that lectin-like oxyLDL receptor-1 (LOX-1) is involved in smooth muscle cell proliferation and neo-intima formation occurring in injured blood vessels. Interestingly, in an experimental model of rat angioplasty, pretreatment with the non-volatile fraction of BEO, reduced the neointima proliferation in a dose-dependent manner, together with free radical formation and LOX-1 expression (Mollace et al., 2008). Moreover, it has been suggested that BEO induces vasorelaxation of the mouse aorta by activating K^+^ channels and inhibiting Ca^2+^ influx (Kang et al., 2013), which differentially modulates intracellular Ca^2+^ levels in vascular endothelial and smooth muscle cells (You et al., 2013). These findings indicate that BEO could be further studied for its potential role as a vasodilator agent in cardiovascular diseases.

## CLINICAL STUDIES

The scientific rationale of essential oils use in aromatherapy to improve mood and the mild symptoms of stress disorders, such as anxiety, depression, and chronic pain, is supported by both the physiological and psychological effects caused by the inhalation of volatile components that are believed to act via limbic system structures, such as the hippocampal formation, the hypothalamus and the pyriform cortex. Indeed, several clinical and experimental data indicate that aromatherapy can improve mood, alertness, and cognition. Specific EEG changes associated with alertness and relaxation have been observed in some studies using essential oils. With regard to BEO, these effects have been attributed to volatile components other than 5-MOP (Bagetta et al., 2010). Although the mechanisms by which BEO induces its effects on the central nervous system have not yet been fully understood, it has been suggested that they could be mediated by the release of amino acids that interact with mechanisms that modulate synaptic plasticity (see *neuropsychopharmacological and neuroprotective activities section*).

Clinical research focused essentially on the therapeutic application of BEO in aromatherapy, by inhalation or hand massage, on anxiety and stress responses. We reviewed the results of ten clinical studies in which only two reported negative results. Their principal data are reported in **Table 1**. Four studies evaluated the effects of different essential oil combinations, with BEO being a constituent of the mixture associated with lavender (*Lavandula angustifolia*), cedarwood (*Cedrus atlantica*), ylang ylang (*Cananga odorata*) or frankincense (*Boswellia carteri*; Graham et al., 2003; Hwang, 2006; Chang, 2008; Hongratanaworakit, 2011). Among the studies with combinations, only Graham et al.’s (2003) study reported negative results. The other three studies showed positive effects based on the subjective responses to stress. The remaining six studies were all conducted using only BEO. Five of these studies reported beneficial effects from BEO, such as reduced heart rate, blood pressure and stress responses (Peng et al., 2009; Seo, 2009; Chang and Shen, 2011; Liu et al., 2013; Ni et al., 2013). One study investigating the effects of aromatherapy with BEO on pain and nausea of children and adolescents undergoing stem cell transplantation, showed no benefit. Unfortunately, most of clinical trials did not report the quality parameters of the essential oils used. Even though these studies are not always satisfactory from a methodological point of view, they show that employment of BEO in aromatherapy can be useful for reducing anxiety and stress responses, and it deserves further clinical investigation.

**Table 1:** Principal characteristics of clinical studies on aromatherapy with bergamot essential oil (BEO), alone or in combination with other essential oils (lavender, cedarwood, ylang ylang, frankincense).

Authors	Study type	Essential oils	Subjects	Principal endpoints	Outcome
Graham et al. (2003)	Double-blind placebo-controlled randomized	Lavender, bergamot, and cedarwood by inhalation	200 patients undergoing radiotherapy	Anxiety measured by HADS	Negative results
Hwang (2006)	Placebo-controlled randomized	Lavender, ylang ylang, and bergamot by inhalation	52 subjects affected by hypertension	Blood pressure, serum cortisol levels, catecholamine levels	Reduction of psychological stress responses, serum cortisol levels, blood pressure
Chang (2008)	Controlled (non-equivalent control group)	Bergamot, Lavender, and Frankincense by hand massage	58 hospice patients with terminal cancer	Pain, depression scales	Reduction of pain and depression
Peng et al. (2009)	Open-label randomized controlled trial	Bergamot by inhalation	114 healthy undergraduate students	Heart rate variability	Reduction of heart rate
Seo (2009)	Placebo-controlled, cross-over	Bergamot by inhalation	36 female high school students	Blood pressure, pulse rate, IgA levels	Reduction of blood pressure and heart rate, no effects on IgA levels
Hongratanaworakit (2011)	Controlled, randomized	Bergamot, lavender cutaneous application	40 healthy subjects	Blood pressure, pulse rate, breathing rate, and skin temperature, recorded as indicators of the arousal level of the ANS	Decrease of subjective behavioral arousal
Chang and Shen (2011)	Not controlled	Bergamot by inhalation	54 elementary school teachers	Blood pressure, heart rate	Decreases in blood pressure and heart rate
Ndao et al. (2012)	Double-blind, placebo-controlled randomized	Bergamot by inhalation	40 children/adolescents to undergo Stem cell transplantation	Pain and nausea scales	No benefit
Ni et al. (2013)	Placebo-controlled, randomized	Bergamot by inhalation	116 patients awaiting ambulatory surgery	Responses to the (STAI) score and vital signs	Reduction of anxiety, heart rate, variability and blood pressure
Liu et al. (2013)	Placebo-controlled, no randomization	Bergamot by inhalation	29 elementary work stressed schoolteachers	Blood pressure, heart rate	Reduction of heart rate

*HADS, Hospital Anxiety and Depression Scale; ANS, autonomic nerbous system; STAI, State Trait Anxiety Inventory; IgA , Immunoglobulin A*.

## PRE-CLINICAL SAFETY OF BEO

Bergamot essential oil is a widely used aromatic ingredient in cosmetics that may be applied on sun-exposed skin areas, although components such as bergapten, citropten, bergamotene, and other furocoumarins may cause phototoxic effects (Chouchi et al., 1996; Kejlovà et al., 2007). Indeed, psoralen can induce skin cancer due to the formation of covalent DNA adducts by exposure to ultraviolet A or solar light (Bakkali et al., 2008). On the contrary, it was reported the photochemoprotection from UVR-induced DNA damage by bergapten (Chadwick et al., 1994). Primarily due to the presence of psoralens, BEO preparations may pose phototoxic, genotoxic and carcinogenic risks. However, a layer of oil on the skin has no sensitizing effect, if not rubbed in, and it has been showed as BEO alone does not irritate the skin (Oppenheim, 1947). Moreover, Trombetta et al. (2010) studied the genotoxic activity of ethanolic bergamot extracts in the SOS (group of cellular functions) chromotest (a bacterial test for detecting DNA-damaging agents), which employs the error-prone DNA repair pathway of *E. coli* PQ37. The experimental results suggested that the extracts, used at doses up to 50 μg/assay, do not induce genotoxicity, even when undergoing metabolic activation. However, in order to guarantee safety, bergapten and other phototoxic components should be removed from BEO, thus resulting a furocoumarin-free BEO. Its use in cutaneous preparations can be considered safe, having no risk of such skin reactions (Belsito et al., 2007). However, safety issues are not separate from the quality aspects regarding the purity of the essential oil or the presence of adulterants. Analysis of BEO authenticity can be performed using enantioselective gas-chromatography (Schipilliti et al., 2011).

## ADVERSE EFFECTS

Despite its wide application, there are only a few reports of phototoxic reactions caused by aromatherapy with BEO. Freund, in 1916, was the first to describe a series of four cases of intense pigmentation in irregular areas after the use of Eau de Cologne before exposure to sunshine. He observed the same phenomenon after experimental use of BEO, one of the constituents of Eau de Cologne. Rosenthal first used the term “Berloque dermatitis” because of the form of the resulting pigmentation (Oppenheim, 1947). Hyperpigmentation of the neck, face, arms, or trunk that have been exposed to light has been attributed to psoralens in the BEO. Cases have become much more rare since the introduction of psoralen-free BEO.

Bergamot essential oil rich in 5-MOP has been used as sun tanning products for many years. In 1995, this use was banned and limited to the treatment of certain skin disorders. Toxic reactions can occur due to psoralen or UVA overdosing or due to accidental exposure to additional UVA, including sunlight exposure. Skin application of psoralens is more likely to induce photosensitivity reactions. To avoid the phototoxic and photocarcinogenic harm, the International Fragrance Association (IFRA) recommended that products that are applied in areas of skin exposed to the sun not have to contain more than 0.4% of BEO.The occurrence of second-degree skin burns was found in two women after PUVA exposure (Herr et al., 2007).

The cases of the two patients were described as having localized and disseminated bullous phototoxic skin reactions that developed 48–72 h after ultraviolet exposure subsequent to aromatherapy with BEO. The first case was of a woman that had used a bergamot aromatherapy oil preparation 3 days earlier and subsequently stayed exposed to the sun for several hours. In the second case, the woman visited a sauna 2 days before, where she was exposed to aromatherapy with BEO before being exposed to UVA radiation while tanning. Cutaneous lesions developed gradually within 48–72 h. In both cases, the patients did not use other creams and/or medications at the same time (Kaddu et al., 2001).

In another case, a 44-year-old man drank up to 4 L of black tea every day for 25 years. When he changed his brand because of occasional gastric pain, he began to drink the same daily quantity of Earl Gray tea. One week after the change, he noticed repeated muscle cramps. After 5 weeks of drinking the tea, the muscle cramps continued. Occasionally, he observed fasciculations, distal paraesthesia, and feelings of pressure in his eyes that were associated with blurred vision. Neurological examination confirmed reduced visual acuity and fasciculations. After 5 months, suspecting a relation between his symptoms and his tea intake, he stopped drinking Earl Gray, going back to black tea. Within 1 week, the symptoms completely disappeared. Successively, he observed that the symptoms did not appear if he ingested no more than 1 L of Earl Gray tea each day. Earl Gray tea is composed of black tea and BEO. The adverse effects of BEO in this patient could be explained by the potential effects of bergapten as a selective axolemmal potassium channel blocker, reducing potassium permeability at the nodes of Ranvier in a time-dependent manner. This may provoke hyperexcitability of the axonal membrane and phase alterations of potassium currents, producing fasciculation and muscle cramps. Impairment of the potassium channel function plays a pathogenic role in other disorders characterized by the occurrence of fasciculation. Prolonged opening of voltage-gated sodium channels caused by bergapten may enhance neuronal hyperexcitability (Finsterer, 2002).

## CONCLUSION

Bergamot essential oil has been traditionally used in Italian folk medicine for magisterial, handcrafted, and homemade preparations that are intended for topical use as antiseptics for the disinfection of skin and as aids for healing minor wounds. BEO is generally well tolerated, but it possesses photosensitive properties because of the presence of furocoumarins, especially 5-MOP. Therefore, in topical preparations, psoralen-free essential oil was used in recent decades. As a consequence of this and because of safety concerns related to furocoumarins, the use of high quality controlled psoralen-free BEO is recommended as a general precaution. However, although the oil has been used extensively for many years, there have only been a few reports of phototoxic reactions to bergamot aromatherapy oil.

Several biological activities of BEO were shown, such as antimicrobial, anti-inflammatory, antiproliferative, and analgesic effects, including effects on the central nervous and cardiovascular systems. Even though these effects indicate potential clinical applications for BEO in the future, to date, only clinical studies investigating aromatherapy effects have been published. The latter were carried out primarily to investigate anxiolytic effects and the reduction of stress responses. They indicate that treatment with BEO in aromatherapy can be useful to reduce anxiety and stress effects.

## Conflict of Interest Statement

The authors declare that the research was conducted in the absence of any commercial or financial relationships that could be construed as a potential conflict of interest.
